# Monitoring Training Loads and Perceived Stress in Young Elite University Athletes

**DOI:** 10.3389/fphys.2019.00034

**Published:** 2019-01-29

**Authors:** Michael John Hamlin, Danielle Wilkes, Catherine A. Elliot, Catherine A. Lizamore, Yaso Kathiravel

**Affiliations:** ^1^Department of Tourism, Sport and Society, Lincoln University, Lincoln, New Zealand; ^2^Sports Doctors, Christchurch, New Zealand

**Keywords:** student-athletes, academic stress, athletic performance, injury, athlete monitoring, illness, sport training

## Abstract

With increased professionalism in sport there has been a greater interest in the scientific approach to training and recovery of athletes. Applying appropriate training loads along with adequate recovery, is essential in gaining maximal adaptation in athletes, while minimizing harm such as overreaching, overtraining, injury and illness. Although appropriate physical stress is essential, stress for many athletes may come from areas other than training. Stress from may arise from social or environmental pressure, and for many athletes who combine elite athletic training with university study, academic workloads create significant stress which adds to the constant pressure to perform athletically. This research aimed to determine if subjective stressors were associated with counterproductive training adaptations in university athletes. Moreover, it aimed to elucidate if, and when, such stressors are most harmful (i.e., certain times of the academic year or sports training season). We monitored subjective (mood state, energy levels, academic stress, sleep quality/quantity, muscle soreness, training load) and objective (injury and illness) markers in 182 young (18–22 years) elite athletes over a 4-year period using a commercially available software package. Athletes combined full-time university study with elite sport and training obligations. Results suggest athletes were relatively un-stressed with high levels of energy at the beginning of each university semester, however, energy levels deteriorated along with sleep parameters toward the examination periods of the year. A logistical regression indicated decreased levels of perceived mood (0.89, 0.85–0.94, Odds Ratio and 95% confidence limits), sleep duration (0.94, 0.91–0.97) and increased academic stress (0.91, 0.88–0.94) and energy levels (1.07, 1.01–1.14) were able to predict injury in these athletes. Examination periods coincided with the highest stress levels and increased likelihood of illness. Additionally, a sudden and high increase in training workload during the preseason was associated with an elevated incidence of injury and illness (*r* = 0.63). In conclusion, young elite athletes undertaking full-time university study alongside their training and competition loads were vulnerable to increased levels of stress at certain periods of the year (pre-season and examination time). Monitoring and understanding these stressors may assist coaches and support staff in managing overall stress in these athletes.

## Introduction

The last 30 years has seen an increase in professionalism in sport and with that has come greater interest in the scientific approach to training and recovery of elite athletes. For athletes, the balance between stress and recovery is crucial for improving sport performance (Kellmann, 2010). On the one hand, adequate physical stress is required in the form of training load which produces fatigue resulting in adaptation of the various bodily systems (Smith, 2003). On the other hand, recovery from training stress is also important if fatigue is to be overcome, adaptation optimized and subsequent performance enhancement realized. Because training, and therefore adaptation, and subsequent performance will be compromised if this balance is not maintained, monitoring of stress and recovery in athletes is vital.

In addition to training loads, elite athletes typically encounter stress from other sources such as social, work-related, lifestyle and athlete-coach relationships. Pioneering work by Pierce and Stratton (1981), suggest that young athletes experience the highest stress when they perform poorly, make mistakes, and when they perceive pressure from parents, coaches, and teammates (Pierce and Stratton, 1981). Athletes who are also involved in university study are very prone to study-related stressors such as coursework demands, study/life balance, and financial strain (Stallman and Hurst, 2016).

When stress (psychological, academic, training, or performance-related) overloads an athlete’s stress-coping ability, the susceptibility to performance decrement increases, as does the risk of injury and illness. The “Stress and Injury” model proposed by Williams and Andersen has been used to explain this relationship (Andersen and Williams, 1988; Williams and Andersen, 1998). According to the model the stress response increases general muscle tension in the body, which can result in reduced motor coordination and flexibility, both of which can influence fatigue. The model also suggests that stress may diminish the visual field, thereby reducing visual attention which may decrease the ability to use relevant peripheral information (Williams and Andersen, 1998). There is a strong body of evidence indicating that an increase in psychosocial stress also increases injury risk in athletes (Williams, 2001; Galambos et al., 2005; Pensgaard et al., 2018), and reducing such stress (via stress-management interventions) decreases the likelihood of injury (Perna et al., 2003). Similarly, chronic high training load stress (Drew and Finch, 2016) or sudden and severe increases in training load stress over a short period of time (Gabbett et al., 2014; Hulin et al., 2014, 2015) can result in significantly higher risk of injury.

Excessive stress (training- and non-training related) not only increases the risk of injury but also the development of acute illness (Walsh et al., 2011b; Gleeson and Pyne, 2016) as well as the risk of overtraining or burnout (Kellmann et al., 2018). Fry et al. (1991) suggested the relationship between health and loading/stress can be viewed as a continuum where load/stress and recovery are the two competing factors ultimately influencing health (Fry et al., 1991). The theory suggests training (and non-training) related loads create stress on the athlete which shifts the athletes psychological and physical well-being along a continuum that advances from homeostasis to acute fatigue, over-reaching, overtraining, subclinical changes (tiredness, lethargy, etc.), clinical symptoms (compromised immunity, influenza, etc.) and illness (or injury). The ideal amount of stress should progress the athlete from the area of homeostasis on the continuum into the area of acute fatigue or over-reaching. However, when adequate recovery is provided, the process is reversed, resulting in adaptation and restoration of homeostasis at a higher level of fitness. Too much stress or inadequate recovery will prohibit adaptation, leading the athlete into the unhealthy and potentially harmful end of the continuum. Monitoring of training- and non-training related stress can therefore enhance the understanding of the training and stress response and help prevent the risk of maladaptation to training which may result in illness or injury (Foster, 1998; Halson, 2014).

Athletes experience stress and subsequent fatigue on a regular basis, yet it is a complicated process (Naokes, 2012), which can follow an individualized pattern unique to each athlete (Mann et al., 2014). Thus monitoring the individual stress response to training and competition is necessary to maintain the unique balance required for homeostasis in each athlete. Such monitoring can take the form of subjective and objective measures used to indicate training load (training volume/duration, rating of perceived exertion, GPS, etc.) and stress/fatigue (perceptual wellness scales, biochemical markers, immu-nological markers, sleep quantity and quality, etc.) (Halson, 2014). Since it is impractical and expensive to monitor large numbers of athletes in the lab, many coaches and trainers have adopted subjective measurement systems to monitor the stress and fatigue of their athletes (Saw, 2015; Nässi et al., 2017). The subjective reporting of training load, perceived stress, and psychological mood states can be a reliable indicator of training load (Robson-Ansley et al., 2009), and can be more responsive to tracking the training response than objective measures (Saw et al., 2016).

The Lincoln University Sports Scholarship program in New Zealand supports approximately 100 athletes each year in 8–10 major sports. Given that these athletes also undertake university study, any accumulation of unmanaged stress may result in injury, illness or a number of other adverse effects. Time away from training or competition due to illness or injury can elicit major consequences, including rehabilitation costs or adverse social, psychological and economic impacts. Having the athlete’s welfare in mind, a monitoring program was developed to monitor subjective measures of stress and fatigue.

The primary objective of this research was to examine the subjective measures that contribute to the overall stress among young elite athletes in a university educational environment. A secondary objective was to investigate the relationship between subjective measures of stress (including training load) and injury or illness.

## Materials and Methods

### Subjects

**FIGURE 1:**
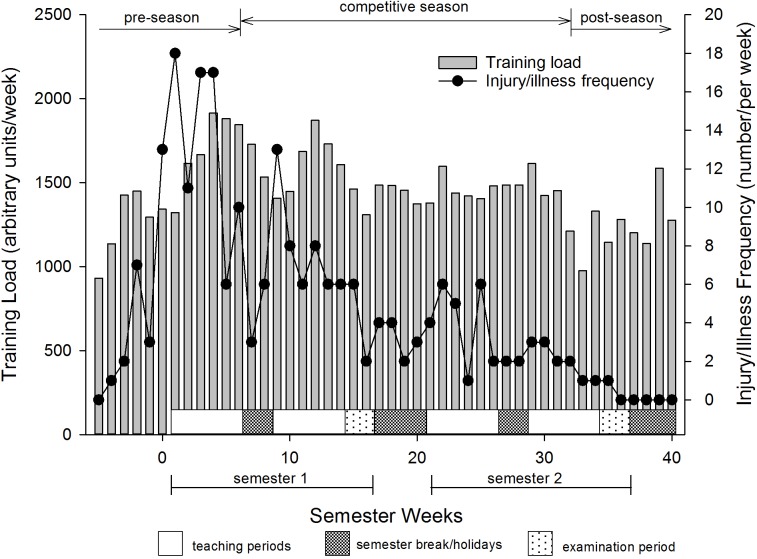
Training loads and injury frequency of young elite university athletes. Values are weekly means.

The perceived stress, training loads and injury/illness incidence were retrospectively investigated from 2014 to 2017 in 182 young athletes during their time at university (approximately-February to October over 4 years). Athletes were involved in a university sport scholarship program where athletes received nutritional, psychological, and medical advice along with individualized training. All participants were young elite athletes (18–22 years old) selected for age-group provincial or national representative honors. This study was carried out in accordance with the recommendations of the Lincoln University Human Ethics Committee. All subjects gave their written informed consent in accordance with the Declaration of Helsinki. The protocol was approved by the University’s Human Ethics Committee (Reference No. 2018-01). Participant characteristics are presented in Table 1. The wide cross-section of athletes sampled caused difficulty in splitting the year of competition into appropriate training phases, however 75% of the athletes represent sports that are played in winter and have a similar training and competitive season. Thus, we have classified the data into the following phases; pre-season (up to and including week 6 of semester 1); competitive season (weeks 7 to 32); and post-season (week 33+) (see Figure 1).

**Table 1:** Characteristics of athletes.

	*n*	Weekly training	Weekly training
		duration (min)	load (arbitrary units)
Male	132	280 ± 184	1557 ± 1046
Female	50	258 ± 175	1486 ± 1040
Rugby	60	294 ± 165	1596 ± 973
Netball	13	247 ± 159	1690 ± 1108
Hockey	35	226 ± 161	1319 ± 972
Cricket	21	219 ± 138	1378 ± 820
Basketball	20	268 ± 169	1536 ± 1083
Rowing	10	295 ± 227	1928 ± 1529
Athletics	4	531 ± 255	1721 ± 874
Football	8	279 ± 195	2037 ± 1611
Other sports	11	142 ± 87	970 ± 585

*Data are mean ± SD. Training load = training intensity (measured via rating of perceived exertion) × training duration (minutes). Other sports included athletes like cyclists, triathletes, throwers, etc.*

### Study Design

This longitudinal retrospective cross-sectional study used a commercially available software system (Health and Sport Technologies, Ltd., trading as Metrifit, Millgrange, Greenore, Co., Louth, Ireland) to collect training data along with subjective feelings of stress, fatigue, academic pressure, mood, sleep quality/quantity as well as clinically-derived incidence of injury or illness in athletes during their time at university. The data was collected using the Metrifit phone application 3–4 weeks prior and then throughout the athlete’s academic year (two semesters). Each semester comprised of 12-weeks of teaching, 1 week of study break, followed by a 2-week final examination period to close the semester. Each semester is interrupted mid-way with a 2-week holiday break. There is a 4-week break between semester 1 and 2, and a summer holiday of 15 weeks prior to the start of the next university year in February. Most students spend holidays (mid and end-of-semester) away from university, for example, returning home to spend time with their families or traveling.

### Training

Every year, individualized training programs were developed by the strength and conditioning staff at the university for each athlete, depending on the type of athlete, their competitive season and injury status. In most weeks, athletes would have at least three training sessions, one sport-specific skills session and one practice game or competition. Athletes recorded their daily training information including type, duration and intensity of training. The intensity of training was estimated using a modified 10-point scale (Foster et al., 2001). Previous research by our group (Hamlin and Hellemans, 2007) and others (Eston and Williams, 1988; Impellizzeri et al., 2004; Gabbett and Domrow, 2007), support these effort ratings as reliable indicators of exercise intensity.

The training load (internal training load) was calculated as the product of volume (duration of training) and intensity (subjective rating of training intensity) as proposed by Foster et al. (2001). It is well-documented that subjective measures (mood disturbance, perceived stress, sleep disruption, etc.) consistently show superior responsiveness to training compared to objective measures (Verde et al., 1992; Coutts et al., 2007; Saw et al., 2016). Unfortunately many existing subjective questionnaires (e.g., Recovery Stress Questionnaire for Athletes (Kellmann and Kallus, 2001), Daily Analysis of Life Demands of Athletes (Rushall, 1990), and Multi-Component Training Distress Scale (Main and Grove, 2009) are long with numerous questions making them time-consuming and complicated and not fit for purpose in a practical setting. Because of this, the Lincoln University Sport Scholarship program decided to incorporate elements of established measures into our own customized, brief, easy-to-use, self-report measure. For this study we asked a series of questions used successfully in a number of other studies (Hamlin and Hellemans, 2007; Hamlin et al., 2017) which were modeled on previous research (Mackinnon and Hooper, 1996; Killen et al., 2010). The questions used in the phone App were based on a five-point Likert scale to record athletes subjective ratings of mood (1 = very stressed, 2 = quite stressed, 3 = slightly stressed, 4 = little stress, 5 = no stress), sleep quality (1 = poor, 2 = below average, 3 = normal, 4 = good, 5 = very good), energy levels (1 = extremely low, 2 = very low, 3 = low, 4 = normal, 5 = high/excellent), muscle soreness (1 = extremely sore, 2 = very sore, 3 = quite sore, 4 = mild soreness, 5 = no soreness), and academic pressure (1 = academic pressure high, 2 = academic pressure building, 3 = heavy academic day, 4 = normal academic pressure, 5 = no academic pressure). In the phone App, athletes had to move an electronic slider (which was initially situated on the far left of the screen, or at number “1” for each question) to the appropriate perceived subjective rating for the day for that question. Athletes also recorded their perceived sleep duration in hours and minutes. The Metrifit software also produces a calculated variable called the Readiness to Train (RTT) score. This variable gives a score out of 100, that is thought to represent the overall stress in the athlete and the estimated ability of the athlete to be ready to train (100 = fully fresh with no fatigue and optimally prepared to train). The RTT score uses the athlete’s subjective measures of mood state, sleep quality, energy level, muscle soreness, academic stress and then applies a weighting appropriate to each subjective measure’s influence on performance and recovery and calculates the RTT. The exact weighting and algorithm used is considered intellectual property (IP) by the software owners and subjective to IP laws. All athletes were given clear instructions on how to use the Metrifit system which included a 2-h training session around understanding the data required by the system and how to enter the data using the Metrifit App Interface on each student’s phone. Athletes were encouraged to use the software to input data daily and they received text message reminders on their mobile phones if data entry was missed.

It is important to not only focus on current training regimes, but also what athletes have previously completed in terms of preparation for training. Previous work suggests a sharp increase in current training (acute training load), without the appropriate preparation (chronic training load), can result in injury (Gabbett, 2016). We therefore calculated the acute:chronic workload which gives an estimate of the preparedness of athletes to handle increases in workload stress using an exponentially weighted moving average (EWMA) as proposed by Williams et al. (2017). The calculation is as follows:

(1) $${\text{EWMA}}_{\text{today}}={\text{Load}}_{\text{today}}{\text{ x λ}}_{\text{a}}+((1-{\text{λ}}_{\text{a}}){\text{ x EWMA}}_{\text{yesterday}})$$

Where λ_a_ is a value between 0 and 1 representing the degree of decay, which assigns a lower weighting for older observations. The λ_a_ was calculated as:

(2) $${\text{λ}}_{\text{a}}=2/(\text{N}+1)$$

Where N is the chosen time decay constant in days, which was selected as 1-week (to represent acute workload over the last 7 days) and 4-weeks (representing chronic workload over the last 28 days). After arbitrarily recording the first observation in the dataset as the first observation, the above formula was used to calculate the average acute and chronic workloads for each week for all subjects combined. The acute:chronic ratio was then calculated by dividing the acute workload by the chronic workload (Williams et al., 2017).

### Injury and Illness

The Metrifit system allows the entry of injury and illness data by the athlete, coach or medical staff. For this study we have used the injury definition of Timpka et al. (2014) which states an injury is a physical complaint or observable damage to body tissue produced by the transfer of energy experienced or sustained by an athlete during training or competition, regardless of whether medical attention was received. In this study most injuries (70%) were diagnosed by a registered physiotherapist or medical doctor. Injuries were counted only once and any re-injury of a previous injury was not included in the data. Injuries were grouped by anatomical location, and nature of injury (e.g., strain, sprain, rupture, etc.) according to current guidelines (Timpka et al., 2014). We also categorized injuries according to the occasion (i.e., match, training), and whether the injury mechanism was via contact or not. Illness was defined as a physical or psychological complaint or manifestation by an athlete not related to injury, causing an impairment in competition or training regardless of whether the athlete received medical attention (Timpka et al., 2014).

### Statistical Analysis

Changes in the mean of the variables and standard deviations representing the between-and within-subject variability were estimated using a mixed modeling procedure (Proc Mixed) in the Statistical Analysis System (Version 9.3, SAS Institute, Cary, NC, United States). Chances that the true effects were substantial were estimated when a value for the smallest worthwhile effect was entered into the calculation. We chose 0.20 standardized units (representing change in mean divided by the between-subject SD at baseline) as the smallest worthwhile change (Cohen, 1988). To make inferences about the true (population) uncertainties in the estimate of change were presented as 90% confidence intervals and as likelihoods that the true value of the effect was increased, decreased or trivial. The descriptors: increased, trivial or decreased were used to describe the direction of the change. Where the confidence interval spanned all three possibilities (increased, trivial, and decreased), the result was deemed unclear. In all other cases, such as no overlap, or an overlap between two possibilities (trivial and increased, or trivial and decreased) a clear result was achieved. The magnitude or probability of the change was assessed using a qualitative scale defined as: <0.5%: almost certainly not; <5%: very unlikely; <25%: unlikely/probably not; 25–75%: possibly, possibly not; >75%: likely, probably; >95%: very likely; and >99.5%: almost certainly.

Team training loads and the incidence of injury were analyzed in SAS using the PROC CORR procedure to determine the association between training load and injury prevalence. The weekly training data varied considerably between different stages of training (e.g., the start of the training year compared to the rest of the training year), therefore, the aggregated weekly results were analyzed during what was believed to be the pre-season for most athletes (e.g., up to and including week 6 of semester 1; i.e., weeks −5 to −6) and the competitive season (weeks 7–32). Since this was aggregated data grouped by week, the results could then be applied to the training group in general.

Individual subjective measures (mood state, sleep quality/duration, energy levels, academic stress), along with injury data were modeled together using a single logistic regression model with a binomial distribution (injured, not injured) and logit link function. These data were analyzed in SAS using the PROC LOGISTIC procedure. The summary statistic used for assessing the adequacy of the fitted model (goodness of fit) was the likelihood ratio chi-square. Odds ratios (and 95% confidence limits) were calculated to determine whether changes in subjective measures increased (or decreased) the odds of injury. Unlike the training data, the subjective data represented individuals, therefore the results can be applied to the individual rather than the training group.

Illness data was fitted with a non-linear regression equation (Peak, Gaussian, 3 Parameter [f = a^∗^exp(−0.5^∗^((x-x0)/b)^∧^2)] to smooth the individual illness frequencies and identify periods of highest illness counts.

## Results

### Training Duration and Load

The training loads for the athletes over the academic year are shown in Figure 1. Five weeks prior to starting university the athletes were completing approximately 931 ± 710 (mean ± SD) arbitrary units (au) of training load per week (∼3.3 h in duration) which increased substantially over the next 8–9 weeks to peak at 1916 ± 1229 (an increase of 106%). Training loads were then maintained at about this level throughout semester 1 apart from slight reductions during semester holidays (students not required to stay on campus to train) and examination periods. The overall average training load was lower (0/18/82, chances of positive/trivial/negative differences in training load; *p* = 0.001) in semester two (1409 ± 952 au) compared to semester one (1594 ± 1079 au).

### Subjective Markers

**FIGURE 2:**
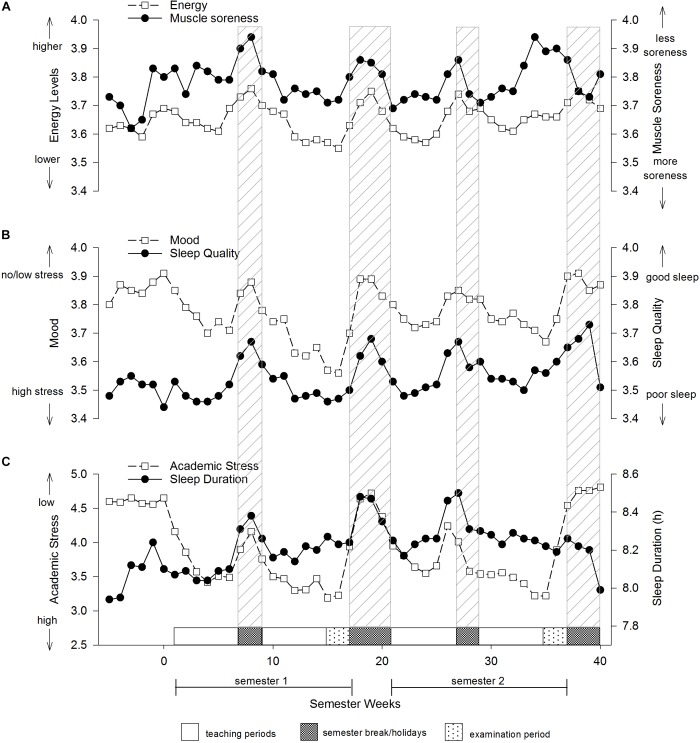
Subjective measures of young elite university athletes. **(A)** Energy and muscle soreness, **(B)** mood state and sleep quality, **(C)** academic stress and sleep duration. Values are weekly means.

At the start of each year, athlete’s perceived energy levels started to decline and only recovered during breaks spent away from university life (Figure 2A). Athletes perceived their energy levels to be lowest during the first semester examination period. Perceived levels of muscle soreness were highest at the beginning of the year, particularly just prior to the start of the teaching. Muscle soreness gradually increased throughout the duration of each semester, but recovered during the mid and end-semester holiday breaks.

Athletes were unstressed at the start of each semester as indicated by their relatively high mood scores (1 = stressed, 5 = unstressed) (Figure 2B). However, as the semester progressed perceived mood scores deteriorated and only recovered back to baseline levels during the holiday breaks. Mood scores were lowest during the two examination periods at the end of each semester, particularly in semester one. Sleep quality mirrored mood scores such that athletes perceived their quality of sleep was highest in the periods away from university and lowest during the examination periods. The sleep quality data was reinforced by the sleep duration data that showed athlete’s sleep duration tended to increase when away from university. Perceived academic stress was at its highest during the examination periods occurring at the end of each semester (Figure 2C).

A relationship was observed between subjective measures of mood, energy, academic stress, sleep duration and the odds of injury, such that lower levels of mood, sleep duration, and academic stress or increased levels of energy were able to predict injury (Table 2). The model was successful at fitting the data as evidenced by the likelihood ratio χ^2^ = 31.76 with 3 degrees of freedom, *p* < 0.001. Converting the odds ratio to percent change [1 – (OR)^∗^100] we found that every unit decrease in mood was associated with a 10.8% increase in the odds of incurring an injury. Similarly, each unit decrease in sleep duration and academic pressure was associated with a 5.9 and 9.0% increase in the odds of getting injured, respectively. Sleep quality was not associated with the odds of injury. The final regression model was (−1.2523 – 0.1137^∗^mood + 0.0056^∗^sleep quality – 0.0605^∗^sleep duration + 0.0706^∗^energy – 0.0947^∗^academic).

**Table 2:** Odds ratios of psychological variables as risk factors for injury in young elite university athletes.

Factor	Odds ratio	95% confidence limits
Mood	0.89^∗^	0.85 to 0.94
Energy	1.07^∗^	1.01 to 1.14
Sleep quality	1.01	0.96 to 1.06
Sleep duration	0.94^∗^	0.91 to 0.97
Academic stress	0.91^∗^	0.88 to 0.94

*^∗^Odds of injury substantially related to factor.*

**FIGURE 3:**
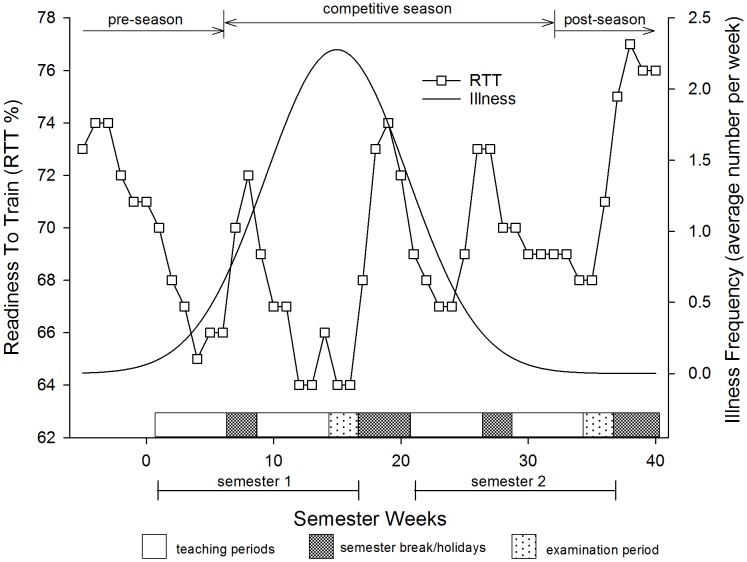
Aggregated ‘Readiness to Train’ variable along with a smoothed regression line for the illness frequencies in young elite university athletes.

Similar to the separate subjective measures, the aggregated RTT score was lowest during the examination periods, particularly in semester 1 (64 ± 15% at week 16, mean ± SD), but recovered to baseline levels (approximately 74–75%) during times when students were away from the university (Figure 3).

### Injury and Illness

The overall incidence of injury and illness for the 45 weeks that athletes recorded their data was 15.6 ± 3.9 injuries per 1000 training hours (mean ± 90% CL). Incidence of injury was higher over the pre-season [up to week 6 (30.7 ± 3.9 injuries per 1000 training hours)], than at any other time during the rest of the year (10.3 ± 1.9 injuries per 1000 training hours), 0/2/98, chances of positive/trivial/negative differences in injury incidence; *p* = 0.01). The majority of injuries sustained over the 4 years were to the ankle and knee which made up almost 30% of all injuries and illness (Table 3). Injuries to the head, shoulder/clavicle, lumbar spine/lower back, thigh, lower leg, and groin were also common. Over half of the injuries sustained over the 4 years were muscle strains and joint sprains (Table 4). The most common illness was lower respiratory tract infection mainly from influenza (Table 3). Illnesses accounted for approximately 14% of the loss of training and playing days and were most notable during times of highest stress which was just before and during semester 1 examinations (Figure 3).

**Table 3:** Location of injury and illness sustained by young elite athletes over 4 years at university.

	*n*	%
Head (including concussion)	15	5.8
Face (including eye, ear, nose)	5	1.9
Shoulder/clavicle	19	7.3
Neck/cervical spine	0	0
Abdomen	3	1.2
Thoracic spine/upper back	6	2.3
Lumbar spine/lower back	14	5.4
Sternum/ribs	4	1.5
Elbow	3	1.2
Upper arm	1	0.4
Finger	4	1.5
Hand	6	2.3
Wrist	5	1.9
Thumb	4	1.5
Thigh	16	6.2
Lower leg	11	4.2
Hip	4	1.5
Groin	11	4.2
Knee	39	15.2
Ankle	36	13.9
Foot/toe	8	3.2
Other	9	3.5
Upper respiratory tract	7	2.7
Lower respiratory tract	19	7.3
Other illness	10	3.9
Total	259	100

*Data are frequencies of injuries and illness and % of total over 4 years.*

**Table 4:** Type and cause of injury sustained by young elite athletes over 4 years at university.

Type of injury	*n*	%
Strain/muscle rupture/tear	78	30.1
Sprain (injury of joint and/or ligament)	64	24.7
Ligamentous rupture	5	1.9
Concussion	16	6.2
Contusion/hematoma/bruise	6	2.3
Fracture (traumatic)	9	3.5
Fracture (stress)	4	1.5
Dislocation/subluxation	7	2.7
Laceration/abrasion/skin lesion	10	3.9
Other	24	9.3
**Cause of injury**		
Contact	101	39.0
Non-contact	110	42.5
Other/missing data (i.e., viral, bacterial)	48	18.5

*Data are frequencies of injuries and % of total injuries over 4 years.*

### Effect of Training Load on Injury Incidence

**FIGURE 4:**
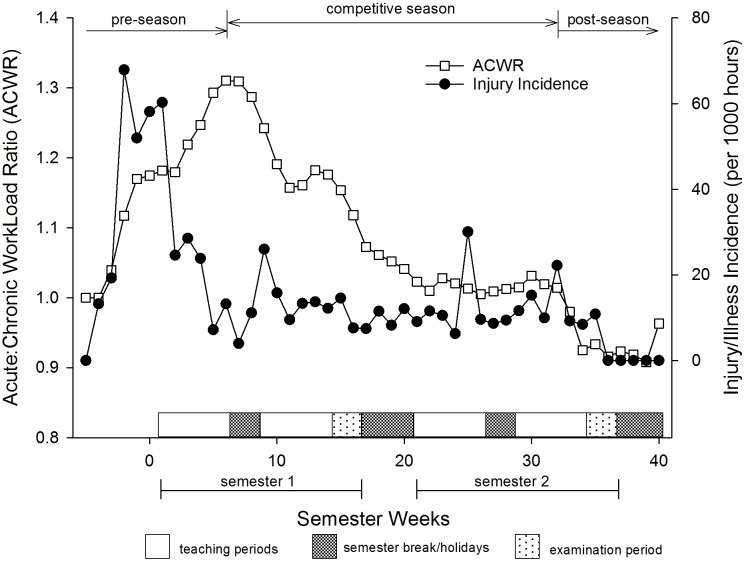
Association between acute:chronic workload ratio and incidence of injury and illness of young elite university athletes. Values are weekly means.

The sudden increase in workload as indicated by the higher acute:chronic workload ratio over the first 9 weeks of pre-season was associated with an increased incidence of injury and illness (*r* = 0.63). During the competitive season, however, increases in training loads resulted in no further increases in the incidence of injury or illness (Figure 1, 4).

## Discussion

The monitoring of athletes has become an important area in sport science, not only because it is the aim of coaches and trainers to give athletes the best care and support, but it is also important to protect athletes as much as possible from any harm or unwanted consequences of training. This research highlights periods within the academic year when athletes undertaking university study are likely to be influenced by increased stress which is associated with increased risk of injury and illness. Our data shows obvious cyclical effects whereby athlete’s subjective measures of stress increased steadily during the semester to reach a nadir during the examination period, after which stress was reduced during the semester break/holiday period (Figure 2).

The overall incidence of injury/illness was ∼16 injury/illness per 1000 training hours over the whole academic year. Since 13% of these were illnesses (mostly influenza) the actual injury rate was slightly lower at 14 injuries per 1000 training hours. This incidence represents the number of injuries that occurred as a result of training and competition combined and is higher than what has been found in football (soccer) (8.0/1000 training hours) (Ekstrand et al., 2011) or rugby league (6.9/1000 training hours) (Killen et al., 2010), but is similar to some earlier research on rugby players (12.4/1000 training hours) (Sparks, 1985). Evidence indicates that stress plays a major part in the etiology of injury (Williams and Andersen, 1997; Rogers and Landers, 2005). Therefore reducing unwanted stress may help reduce the incidence of injury, particularly during the pre-season period in the athletes of this study.

In this study we observed a relationship between a number of subjective measures and odds of injury. In particular, this study showed that mood, sleep duration (but not quality) and academic pressure were the strongest contributors to injury. These findings corroborate previous work by Galambos et al. (2005) who found subjective measures (mood disturbance and increased perceived life stress) were able to predict injury in elite athletes. Moreover, results from this study highlight the importance of measuring subjective stress variables in elite athletes at university. Indeed, a large majority of the models in the sport injury literature suggest that sport injuries result from an accumulation of not just physical but psychosocial stressors (Pensgaard et al., 2018).

Probably the most influential theory on the relationship between psychosocial stress and injury is the early work of Andersen and Williams (1988) who outlined a stress-injury model that suggested athletes who accumulate stress levels that overcome their stress-coping abilities are unable to relax which subsequently alters the athletes attentional ability (Williams et al., 1986). Compromised attention may then result in a failure to detect vital clues about the athletes body or the environment and/or increases in their muscle tension (Nideffer, 1983), thereby disturbing motor coordination and increasing the risk of injury (Andersen and Williams, 1988). Andersen and Williams’ theory implies, that if situational demands exceed an athlete’s coping ability, elevated stress levels will result. The stress response is heightened further if the athlete perceives the consequences of their performance will detrimentally affect the athlete’s sports career or self-esteem (Andersen and Williams, 1988). Indeed, the athletes involved in this study were under considerable stress over and above what normal university students might encounter. The sport scholarship athletes must not only pass their courses throughout their scholarship tenure, but they must also perform to high expectations in the gym (meet strength and conditioning targets) and on the sports field (be selected for certain development or representative teams which compete at the highest level for this age group). This increased stress, particularly at the beginning of the year, in the athletes of this study may have led to the higher rates of injury.

A regression analysis showed that the individual subjective measures (mood state, academic stress, etc.) were related to the risk of injury. When employing an aggregated measure of several subjective variables in the regression model (RTT score), a stronger relationship with the risk of injury emerged (OR = 0.58, CL 0.47 to 0.71). Perhaps analyses using an aggregate variable comprised of a number of subjective measures (e.g., academic, mood, sleep, energy levels) may be better suited to monitoring overall subjective levels of athlete stress than individual subjective measures, particularly when determining relationships with the risk of injury.

Interestingly an increase in subjective feelings of energy was associated with a 7.3% increase in the odds of injury (Table 2). While this seems counter-intuitive, since injury is normally associated with negative emotional states (Kolt and Kirby, 1994), more recent research suggests injury may also be associated with positive emotional states (Hanin, 2000). It has been theorized that being successful or feeling energized may result in complacency, which might lead to a decrease in alertness and a subsequent increased risk of injury (Devonport et al., 2005). On the other hand, being overconfident may also prevent utilization of all resources during activity, resulting in underperformance and injury. Overconfidence may result in risk-taking behavior during training or competition which could also result in increased injury risk. Jones et al. (2017) noted this unexpected relationship and suggested that when athletes perceive themselves to have high energy levels (low fatigue) they may train or play at higher intensities, thereby increasing the forces and strains involved during exercise resulting in higher risk of injury (Jones et al., 2017). Whatever the cause of this association, coaches, and support staff need to be vigilant that athletes follow appropriately-prescribed training loads (even if they have low fatigue levels and are feeling highly energized) in order to avoid injury.

Our study also found that high acute:chronic team training workloads were associated with an increased risk of injury. High acute:chronic workload, particularly at the beginning of the year (during the first 7 weeks where athletes are in their pre-season period), increased the incidence of injury and illness almost fourfold compared to the rest of the year (Figure 4). Gabbett and Domrow (2007) also found increased workload toward the beginning of the training season resulted in increased injury prevalence (Gabbett and Domrow, 2007). Previous research suggest inadequate pre-season training or low off-season aerobic fitness increased athlete’s risk of injury during the pre-season (Gabbett and Domrow, 2005). The reduction in injury and illness despite the increase in workload during the semester (from weeks 7 onwards) would suggest the high injury/illness spike during weeks 1–7 may be due to insufficient preparation prior to commencing pre-season training. Indeed, the highest muscle soreness levels of the year also occurred during this period which would indicate physical unpreparedness of the athletes. These data suggest there is a critical window during the pre-season when coaching and support staff need to be attentive to avoid unwanted illness and injury.

Previous researchers have suggested acute:chronic workloads within the range of approximately 0.8–1.3 represent the ‘sweet spot’ where training load is high enough to result in adaptation but not too high as to cause a heightened risk of injury (Blanch and Gabbett, 2016; Gabbett, 2016). These authors suggested an acute:chronic workload above 1.5 increased the risk of injury in athletes. The average weekly acute:chronic workload in our athletes was consistently below 1.5 (Figure 4) and yet our athletes still incurred injuries. The differences in athletes between studies may account for the higher injury incidence at a lower acute:chronic workload, although Blanch and Gabbett (2016) data was based on team sport athletes who make up a large proportion of the athletes in this study. However, most of the research by Blanch and Gabbett (2016) and Gabbett (2016) was on professional elite athletes who probably have a greater training base than our athletes and can sustain a larger increase in the acute:chronic workload before injuries occurred. Other studies investigating injury prevalence in collision sports suggest the introduction of contact drills and skills into training may increase the risk of injury (Gabbett and Domrow, 2005), however, this is unlikely in this study, since such drills and skills were not introduced until mid-way through the start of semester one when injury incidence was reducing.

The most common injuries tended to be joint injuries (shoulder, knee and ankle made up 36.4% of all injuries) that occurred predominantly during the pre-season period. All athletes were given off-season programs, therefore it was not a matter of inadequate information causing the injuries, but perhaps a lack of motivation. A possible solution might be to incorporate positive reinforcement to encourage athletes to comply with their pre-season training program (e.g., fewer sport scholarship ‘chores’ to attend to if athletes meet certain fitness targets). Perhaps the challenge of a running time trial on their first week back of semester might also encourage maintenance of fitness standards over the off-season break. Perhaps we should anticipate a larger decline in fitness over the off-season and cater for this by having a ‘home-based preseason’ warm-up period which would prepare athletes prior to coming to campus for their actual preseason training. An additional approach could be to introduce stress management skills along with muscle relaxation and attentional awareness techniques that may help reduce stress and thereby vulnerability to injury (Olmedilla-Zafra et al., 2017).

This study found that in times of high stress, illness rates increased substantially, particularly during the winter semester (Figure 3). This trend was most obvious during the end of semester one leading into the first examination period of the year. Periods of heavy training have been linked to depressed immunity and subsequent illness (Walsh et al., 2011a). However, the most dangerous period for illness in our athletes was at a time when training load was relatively moderate (Figure 3), suggesting training stress may not be the sole culprit behind the increased prevalence of illness; a finding common in the literature (Fricker et al., 2005; Veugelers et al., 2016). Previous research indicates that impaired immune function may also be related to sleep deprivation (Simpson et al., 2017). Indeed, an earlier study reported subjects with less than 7 h sleep were almost three times more likely to develop influenza than those sleeping eight or more hours (Cohen et al., 2009). It is also well-known that academic stress (academic tests and papers, etc.) is positively correlated with the occurrence of illness in university students (Lesko and Summerfield, 1989). We postulate that the combined effects of oncoming winter, along with regular training with low sleep quality and reduced sleep duration and high academic stress may have acted to push the athlete along the health continuum away from homeostasis and toward maladaptation thereby suppressing the immune response in our athletes resulting in an increased likelihood of developing illness over the semester one examination period.

This study has three key limitations that should be noted. Firstly, the use of ‘bespoke’ subjective questions used in the phone App questionnaire in this study suggests that results should be considered speculative until substantiated by vigorous validity and reliability testing. Secondly, this research relied on the timely and correct entering of accurate data by the athletes and any deviation from this practice may have corrupted the data. Thirdly, the participants of this study are all young people engaging in elite sports programs and university study, therefore, the results of this research may only apply to this cohort and may not be generalizable.

This is not the first study to investigate the association between subjective markers and injury/illness in athletes. However, several factors make this study unique, including the collection of long-term (over 4 years) subjective data, which provide an overall impression of the change in these measures during the academic year for elite athletes. This study also collected clinically-diagnosed illness and injury data allowing us to investigate the links between injury/illness and subjective levels of stress. Finally, the dataset itself, using young elite athletes undertaking university study is relatively distinctive. Although the study encompasses a broad spectrum of athletes from a wide variety of sports, the findings are quite clear. Athletes undertaking academic workloads in addition to their normal physical training and competition stresses are vulnerable at certain times of the year to increased stress (pre-season and examination time). These results have implications for not only the athletes, but also their coaches, administrators, and other support staff (athletic and academic). The implications are that they may better understand that certain clusters of subjectively-reported stressors can trigger higher amounts of stress, which can lead to increased risk of injury and illness. Moreover, the results suggest adopting a stress-reduction program, particularly prior to the pre-season and examination periods, might help to prevent issues from arising, or help to efficiently mitigate and manage those that do arise.

## Data Availability Statement

The dataset for this manuscript is not publicly available because of commercial sensitivity in regards to the software system used in collecting the data (Health and Sport Technologies, Ltd., trading as Metrifit, Millgrange, Greenore, Co., Louth, Ireland). Requests to access the datasets should be directed to MH mike.hamlin@lincoln.ac.nz.

## Author Contributions

MH conceptualized and designed the study. MH and DW assisted in the planning and acquisition of data. MH, DW, CE, CL, and YK helped with the analysis and interpretation of the data, critically revising the manuscript, and adding important intellectual content. All authors gave approval for the final version of this manuscript to be published and agreed to be accountable for all aspects of the work.

## Conflict of Interest Statement

YK was employed by company Sports Doctors, Christchurch, New Zealand. The remaining authors declare that the research was conducted in the absence of any commercial or financial relationships that could be construed as a potential conflict of interest.
